# Association Between the Methylation Statuses at CpG Sites in the Promoter Region of the *SLCO1B3*, RNA Expression and Color Change in Blue Eggshells in Lushi Chickens

**DOI:** 10.3389/fgene.2019.00161

**Published:** 2019-02-26

**Authors:** Zhuanjian Li, Tuanhui Ren, Wenya Li, Yu Zhou, Ruili Han, Hong Li, Ruirui Jiang, Fengbin Yan, Guirong Sun, Xiaojun Liu, Yadong Tian, Xiangtao Kang

**Affiliations:** ^1^College of Animal Science and Veterinary Medicine, Henan Agricultural University, Zhengzhou, China; ^2^Henan Innovative Engineering Research Center of Poultry Germplasm Resource, Zhengzhou, China

**Keywords:** methylation, *SLCO1B3* gene, promoter, blue eggshell, chicken

## Abstract

The formation mechanism underlying the blue eggshell characteristic has been discovered in birds, and *SLCO1B3* is the key gene that regulates the blue eggshell color. Insertion of an endogenous retrovirus, EAV-HP, in the *SLCO1B3* 5′ flanking region promotes *SLCO1B3* expression in the chicken shell gland, and this expression causes bile salts to enter the shell gland, where biliverdin is secreted into the eggshell, forming a blue shell. However, at different laying stages of the same group of chickens, the color of the eggshell can vary widely, and the molecular mechanism underlying the eggshell color change remains unknown. Therefore, to reveal the molecular mechanism of the blue eggshell color variations, we analyzed the change in the eggshell color during the laying period. The results indicated that the eggshell color in Lushi chickens can be divided into three stages: 20–25 weeks for dark blue, 26–45 weeks for medium blue, and 46–60 weeks for light blue. We further investigated the expression and methylation levels of the *SLCO1B3* gene at eight different weeks, finding that the relative expression of *SLCO1B3* was significantly higher at 25 and 30 weeks than at other laying weeks. Furthermore, the overall methylation rate of the *SLCO1B3* gene in Lushi chickens increased gradually with increasing weeks of egg production, as shown by bisulfite sequencing PCR. Pearson correlation analysis showed that methylation of the promoter region of *SLCO1B3* was significantly negatively correlated with both *SLCO1B3* expression in the shell gland tissue and eggshell color. In addition, we predicted that CpG5 and CpG8 may be key sites for regulating *SLCO1B3* gene transcription. Our findings show that as the level of methylation increases, methylation of the CpG5 and CpG8 sites hinders the binding of transcription factors to the promoter, reducing *SLCO1B3* expression during the late period and resulting in a lighter eggshell color.

## Introduction

Recent years have seen substantial progress in the field of optimizing poultry science through genetic improvement. Eggs of poultry and other birds can vary widely in terms of coloration and patterning, with colors ranging from brown or white to red, violet, or turquoise, and the potential for speckling and other patterning (Wang et al., 2009). Pyrroles are responsible for this diversity of color, with protoporphyrin and biliverdin being the key known eggshell pigments (Kennedy and Vevers, 1976; Gorchein et al., 2010; Igic et al., 2010). Brown colored eggs arise due to the protoporphyrin pigment alone, while when protoporphyrin and biliverdin are both present eggs can take on a blue color and white eggs can arise when both pigments are present at low levels or none at all (Kennedy and Vevers, 1976).

The coloring of eggshells is a form of crypsis or mimicry developed during evolution that can both strengthen this shell and can help to filter out solar radiation (Underwood and Sealy, 2002). Blue eggshells have been found both among wild birds, including eastern bluebird, blue-footed booby, and pied flycatcher (Moreno et al., 2005; Siefferman et al., 2006; Morales et al., 2010), as well as among domestic birds, including chickens, ducks and Japanese quail (Ito et al., 1993; Wang et al., 2009; Liu et al., 2010). Brown, white, pink and blue are the four most common eggshell colors in chickens, with brown and white representing the two major colors. Biliverdin, biliverdin zinc chelate, and protoporphyrin-IX are the three main pigments of eggshell color (Lang and Wells, 1987). Eggshell color is an important indicator for assessing egg quality characteristics, as the degree of color uniformity can reflect the level of production consistency and the purity of the breed (Li et al., 2003). A range of breeds known to lay blue eggs have been identified, including the Chilean Araucana breed and the native Chinese Dongxiang and Lushi chickens, whose blue eggs exhibit dominant inheritance (Wang et al., 2013). The Lushi chicken is one of the native chicken breeds of Lushi County in Henan Province of China. The unique geographic environment provides a relatively wide range of foraging habitats for the Lushi chicken. This breed can lay blue eggs and has superior meat quality.

Eggshell color is a qualitative trait controlled by specific modified genes, and breeders have carried out many studies on the blue eggshell regulatory mechanism. The oocyan gene, mediating eggshell bilverdin accumulation, was first reported to be autosomal dominant in chickens, resulting in blue-shelled eggs (David et al., 2013). A previous report indicated that insertion of an endogenous retrovirus, EAV-HP, in the 5′ flanking region of *SLCO1B3* promotes the expression of the *SLCO1B3* gene (GenBank Accession No. NM_001318449.1) in the uterus of the oviduct and causes blue eggshells in chickens (Wang et al., 2013). To date, the formation mechanism underlying the blue eggshell character has been elucidated, and *SLCO1B3* is the key gene that regulates the color of the blue eggshell (David et al., 2013).

*SLCO1B3* was determined to be the only gene uniquely expressed in the uterine tissue of blue-shelled but not non-blue-shelled hens. The genetic pattern of blue eggshells in ducks are comparable to those in chickens, with a single gene-mediated dominant phenotype. The *SLCO1B3* gene, however, does not exhibit expression within the uterine tissue of ducks with blue eggs, and there is no evidence of EAV-HP insertion with the homologous region of these animals. The expression of the *SLCO1B3* gene causes bile salts to enter the shell glands, from where it is secreted into the eggshell, forming a blue shell. Blue eggshell coloration is a trait which is inherited in an autosomal dominant manner, with eggs from heterozygous animals being lighter blue than those from homozygote. *SCLO1B3* is known to be expressed two- to three-fold higher in homozygous Dongxiang and Lushi chickens relative to heterozygous birds (Wang et al., 2013).

Epigenetics modifications affect the expression of genetic material without nucleotide sequence alterations (Holliday, 1987; Bernstein, 2007). These mechanisms are essential throughout the developmental process of organisms, primarily in relation to aging (Jones and Martienssen, 2005). DNA methylation, the most common epigenetic modification, plays central roles in various biological processes, including transcriptional regulation, cell development, genomic imprinting, X chromosome inactivation, transposon silencing, and tumorigenesis (Cedar, 1988; Bird, 2002; Li and Zhang, 2014; Bestor et al., 2015). DNA methylation uses *S*-adenosylmethionine (SAM) as a methyl donor, with DNA methyltransferases (DNMTs) selectively transferring activated methyl groups to cytosine bases, forming 5-methylcytosine (5-mC), primarily in cytosines in CpG dinucleotide-rich islands (Bird, 1986; Sanders and Parker, 2010; Auclair and Weber, 2012). Extent of DNA methylation is often negatively correlated with corresponding gene expression (Mohn et al., 2008; Mendelson et al., 2017). Furthermore, this correlation is multifactorial and involves temporal, spatial and structural regulation (Vivian et al., 2017). While promoter methylation of CpG islands (CGIs) is generally accepted to decrease the transcription of downstream genes, DNA methylation in gene bodies has been discovered to show the opposite trend and is thought to be a consequence of gene expression rather than a cause (Gutierrezarcelus et al., 2013). Moreover, studies have indicated that the degree of gene expression and methylation correlation can be quite modest (Jung et al., 2012; Legendre et al., 2016).

A large body of literature has reported DNA methylation in livestock. One previous study systematically analyzed patterns of promoter methylation in a genome-wide manner in wild and domestic chicken muscle samples. The authors found that changes in CG motifs did not alter the overall methylation patterns in domesticated chickens, and single nucleotide polymorphisms (SNPs) in promoter regions did not alter overall DNA methylation. Even those growth-related genes which were found to have undergone substantial genetic still exhibited similar DNA promoter methylation patterns between animals (Li et al., 2015). A separate recent study examining the DNA methylation and gene expressions of high- and low-altitude Tibetan pigs found that the low-altitude animals exhibited shifts in DNA methylation patterns associated with low-altitude acclimation (Jin et al., 2018).

In the present study, to reveal the molecular mechanism underlying the blue eggshell color variations, we analyzed the change in the eggshell color during the laying period, and the results indicated that the eggshell color in Lushi chickens can be divided into three stages: 20–25 weeks for dark blue, 26–45 weeks for medium blue, and 46–60 weeks for light blue. We further investigated the expression and methylation levels of the *SLCO1B3* gene during eight different weeks, and the results showed that the relative expression level of *SLCO1B3* was significantly higher during weeks 25 and 30 than during other laying weeks (*P* < 0.05) and that the expression level was significantly higher during weeks 35, 40, and 45 than during weeks 50, 55, and 60 (*P* < 0.05). Furthermore, finding that the overall methylation rate of *SLCO1B3* in Lushi chickens increased gradually with increasing weeks of egg production. Pearson correlation analysis showed that the promoter methylation of *SLCO1B3* was significantly negatively correlated with *SLCO1B3* expression in the shell gland tissue and the eggshell color. In general, these data suggest that promoter DNA methylation of the *SLCO1B3* gene might participate in regulating the color change in blue eggshells in chickens.

## Materials and Methods

### Animals

All animal experiments were performed according to Regulation for the Chinese National Research Council (1994). All experimental procedures and methods were approved by the Henan Agricultural University Institutional Animal Care and Use Committee (Permit Number 11-0085). A total of 200 randomly selected 20-week-old Lushi blue-shelled chickens were raised in stair-step cages under identical environments, with feed and drinking water provided *ad libitum* (Ren et al., 2018).

### Measurement of Eggshell Color and Sample Collection

A colorimeter (Type NR-10) (Mingao, Nanjing, China) for eggshell color was used for quantitative measurement of blue-shelled eggs from the 200 chickens in nine different weeks (20, 25, 30, 35, 40, 45, 50, 55, and 60 weeks). During color measurement, data were recorded from normal eggs, whereas rough-shelled eggs and abnormal eggs were excluded. Starting from the obtuse end of the egg, the obtuse, medium and acute ends of the eggshell were measured along the longitudinal axis of the egg (Supplementary Figure S1). From the three measured values for each egg, the mean was computed and represents the eggshell color value for each egg, and the greener the eggshell color, the smaller the measured value. Shell gland tissue samples at 25, 30, 35, 40, 45, 50, 55, and 60 weeks were used; shell gland tissue was taken from five chickens each week and stored in a refrigerator at -80°C until further analysis.

### The Detection of Blue Shell Genotypes

The expression level of *SCLO1B3* gene is two- to three- fold higher in homozygous blue-shelled chickens than in heterozygous blue-shelled Lushi chickens (Wang et al., 2013). Therefore, to ensure the accuracy of the subsequent experimental results, the experiment needed to screen individuals with dominant homozygous traits for blue shells and exclude individuals with heterozygous traits for blue shells. Genomic DNA was extracted from shell gland tissue samples by a DNA extraction kit (TIANGEN, Beijing, China) according to the operating instructions. The specific detection primers for blue shell genotypes were designed by the Henan Research Center of Poultry Germplasm Resource, and the final diluted concentration of all primers was 10 μM (Supplementary Table S1).

### Analysis of *SLCO1B3* Gene Expression in Chickens

We used qRT-PCR to analyze the relative expression levels of *SLCO1B3* in shell gland tissue and at different ages of chicken. The specific primers of *SLCO1B3* gene: (forward) 5′-TGGTGATTGCAT TTGTAAGCTA-3′ and (reverse) 5′-TGGAGAGCAGGGATTTATGC-3′. The *β-actin* was used as a reference gene, and the forward and reverse primer sequences were 5′-GAGAGAAGATGACACA GATC-3′ and 5′-GTCCATCACAATACCAGTGG-3′, respectively. The qRT-PCR product length of *SLCO1B3* gene was 197 bp and that of the *β-actin* gene was 116 bp in the qPCR assay. Total RNA was extracted using Trizol (Invitrogen, Carlsbad, CA, United States), and cDNA was synthesized with RNA reverse transcription kit (Takara, Dalian, China) according to the operating instructions. The qPCR program was carried out in a Roche LightCycler^®^ 96 instrument by the SYBR Green method, and all reactions were three biological replicates. The relative expression level and significance in different developmental periods were analyzed by the 2^-ΔΔct^ method and one-way analysis of variance (ANOVA) (Schmittgen and Livak, 2008; Ren et al., 2017).

### Methylation Template DNA Modification and Purification

DNA samples from eggshell gland tissue were obtained from three individuals each week. The DNA template was treated using a MethylEdge^TM^ Bisulfite Conversion System kit (Promega, Beijing, China) according to the operating instructions.

### The Prediction of CpG Island and Primer Design

The reference sequence for the chicken *SLCO1B3* gene has been published in NCBI (GenBank Accession No. NC_006088.4). The online software MethPrimer^1^ was used to analyze CGI in the chicken *SLCO1B3* gene promoter region and to design specific BSP (bisulfite sequencing PCR) methylation primers. All primers were designed near the EAV-HP insertion region of the *SLCO1B3* gene promoter, and the PCR products were specifically detected using 1.5% agarose gel (Supplementary Table S1).

### PCR Amplification of Modified DNA

Each 50 μL PCR volume contained 2.5 μL template DNA, 2 μL each primer, 25 μL 2×*Taq* Master Mix (Kangwei, Beijing, China), and 18.5 μL ultrapure water. The Touch-Down PCR cycle parameters were as follows: initial denaturation at 94°C for 3 min, followed by 14 cycles of denaturation at 94°C for 30 s; annealing at 65–50 °C for 30 s; and extension at 72°C for 20 s; followed by 30 cycles at 94°C for 30 s; 50°C for 30 s; 72°C for 20 s; with an additional 10 min extension at 72°C (Li et al., 2018). The PCR products were purified and recovered by a DNA Universal DNA Purification Kit (Tiangen, Beijing, China). Next, purified PCR products were cloned into the pMD^TM^18-T vector (Kangwei, Beijing, China) and transformed into *E. coli* DH5α-competent cells (Kangwei, Beijing, China) for further replication, and then positive clones were screened and sequenced (Sangon, Shanghai, China). The pMD^TM^18-T vector was transformed from the pUC18 vector, and the EcoR V recognition site was inserted between the Xba I and Sal I recognition sites at the multiple cloning site of the pUC18 vector. Then, enzyme digestion was accomplished using EcoR V, adding a “T” to the 3′ end on both sides. *E. coli* DH5α-competent cells are a strain commonly used for plasmid cloning.

### Sequence Comparison of *SLCO1B3* and Transcription Factor Prediction

Each week, 12–16 positive clones were screened and sequenced in three eggshell gland tissues (Sangon, Shanghai, China). Sequencing results of the CGI in the *SLCO1B3* gene promoter region were compared with the reference sequence by BioXM software (Version 2.6), and the number of methylation sites was counted. An online tool to calculate the methylation share rate^2^ was used to map the methylation pattern of the *SLCO1B3* gene, and the transcription factors (TFs) in the gene promoter region were predicted using AliBaba software (Version 2.1)^3^. The calculation of the CGI integral methylation rate was as follows: CpG dinucleotide site of the CGI inner methylation as a percentage of the population in the overall sequenced positive clone.

## Results

### Change in Eggshell Color in Different Weeks

According to the data measured in nine different weeks, the average values of eggshell color at 20, 25, 30, 35, 40, 45, 50, 55, and 60 weeks were 53.53, 53.86, 56.14, 56.59, 56.95, 56.88, 58.57, 57.79, and 58.40 in sequence, respectively (Supplementary Table S2). The egg production periods of Lushi chickens could be divided into three stages as follows: 20 to 25 weeks of age for dark blue, 26 to 45 weeks of age for medium blue, 46 to 60 weeks of age for light blue. The measuring range of eggshell color was 48.24–65.55, and the standard deviation range was 2.58–4.07. The variable coefficient of eggshell color at each age ranged from 4.79 to 7.16%, all of which were lower than 10%, indicating that the color uniformity of the eggshells was good. The standard deviation in eggshell color was the highest at 45 weeks of age, with a value of 4.07, indicating that the eggshell color range was large and that its uniformity was relatively poor. The standard deviation in eggshell color was 2.58 at 25 weeks of age, indicating that the color variation range was the smallest and that its uniformity was better (Table 1).

**Table 1:** The statistical analysis of the measured value of eggshell color in each week.

Week	Sample size	Mean	Standard deviation	Maximum	Minimum	Variable coefficient
						
20	264	53.53	2.8	57.31	48.24	0.0524
25	337	53.86	2.58	60.3	49.63	0.0479
30	313	56.14	3.14	62.32	48.43	0.0559
35	290	56.59	2.93	64.02	49.75	0.0517
40	285	56.95	3.33	64.84	48.37	0.0585
45	319	56.88	4.07	64.38	48.77	0.0716
50	348	58.57	2.97	65.3	51.8	0.0507
55	313	57.79	3.48	65.55	50.43	0.0603
60	184	58.4	3.19	64.34	51.66	0.0546


**FIGURE 1:**
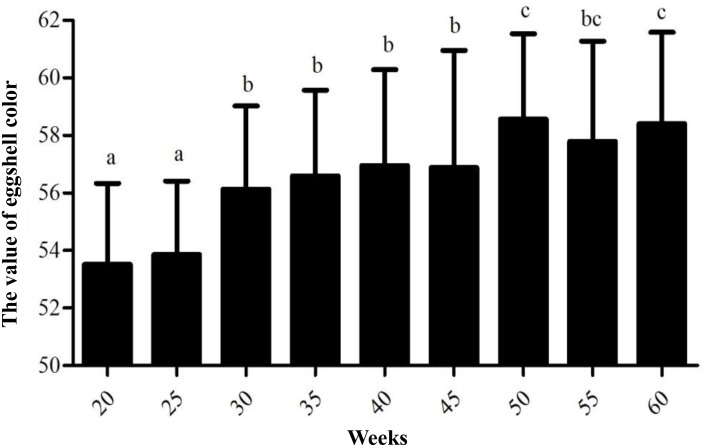
The value of eggshell color at different weeks. Different lowercase letters indicate *P* < 0.05, and the same letters indicate no difference (*P* > 0.05); the larger the eggshell color value is, the lighter the eggshell color of the egg.

Variance analysis of different weeks showed that the eggshell color at 20 and 25 weeks of age was significantly deeper than that of 30, 35, 40, and 45 weeks of age (*P* < 0.05) and that the eggshell color was not significantly different at 30, 35, 40, and 45 weeks of age (*P* > 0.05). Furthermore, the eggshell color at 50 and 60 weeks of age was significantly lighter than that at the previous 30, 35, 40, and 45 weeks of age (*P* < 0.05). However, the color at 55 weeks of age became lighter than that at 50 and 60 weeks old, but the difference was not significant (Figure 1). During the laying period, the eggshell color values of the Lushi chicken were mainly concentrated between 50 and 62 (Supplementary Table S2).

### Determination of Blue-Shelled Egg Genotypes

The shell gland tissue genomic DNA from different chicken breeds was amplified using the blue shell detection primer, and the PCR products were genotyped using 1.5% agarose gel electrophoresis. Fragment lengths of 776 bp indicated dominant homozygous, fragment lengths of 776 and 495 bp indicated dominant heterozygous, and fragment lengths of 495 bp indicated recessive homozygous (Supplementary Figure S2). The results of the genotyping analysis of genomic DNA samples from shell gland tissue indicated that all of the included Lushi chickens were homozygous for the blue shell genotype (Supplementary Figure S2).

### Expression of *SLCO1B3* and Correlation Analysis With Eggshell Color

**FIGURE 2:**
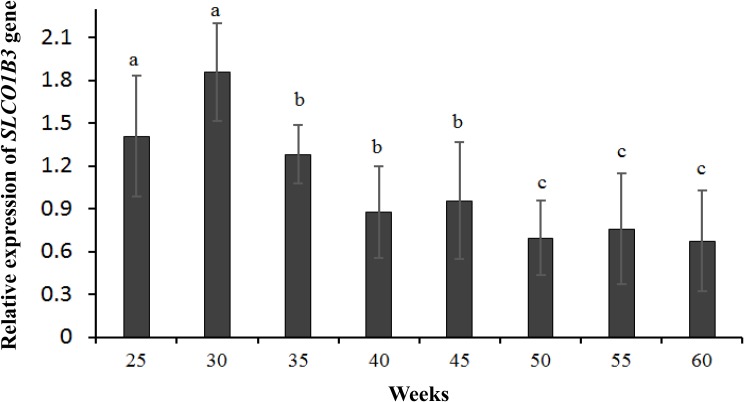
The expression of the *SLCO1B3* gene in the shell gland of Lushi blue eggshell chickens at different weeks. Different lowercase letters indicate *P <* 0.05, and the same letters indicate no difference (*P >* 0.05).

**FIGURE 3:**
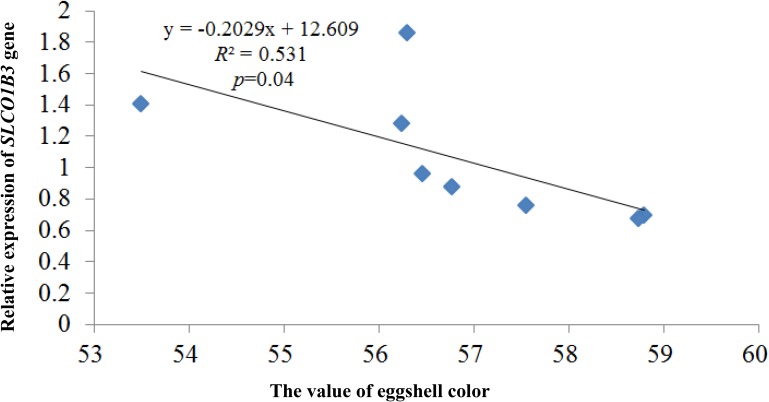
The correlation analysis between the expression of the *SLCO1B3* gene and the value of eggshell color. The larger the eggshell color value is, the lighter the eggshell color of the egg.

qPCR indicated that the expression of *SLCO1B3* at different developmental stages was different in the shell gland tissue. The relative gene expression levels at weeks 25 and 30 were significantly higher than those at other weeks (*P* < 0.05), and the gene expression levels were significantly higher at weeks 35, 40, and 45 than at weeks 50, 55, and 60 (*P* < 0.05). Moreover, gene expression did not differ significantly among weeks 35, 40, and 45 (*P* > 0.05), nor among weeks 50, 55, and 60 (*P* > 0.05) (Figure 2). According to the Pearson correlation analysis, a strong positive correlation existed between the average values of eggshell color and *SLCO1B3* expression in the shell gland in nine different weeks (*P* < 0.05); the correlation coefficient between the eggshell color and gene expression was calculated to be 0.531 (Figure 3).

### The Analysis of *SLCO1B3* Gene Methylation and BSP Sequence

**FIGURE 4:**
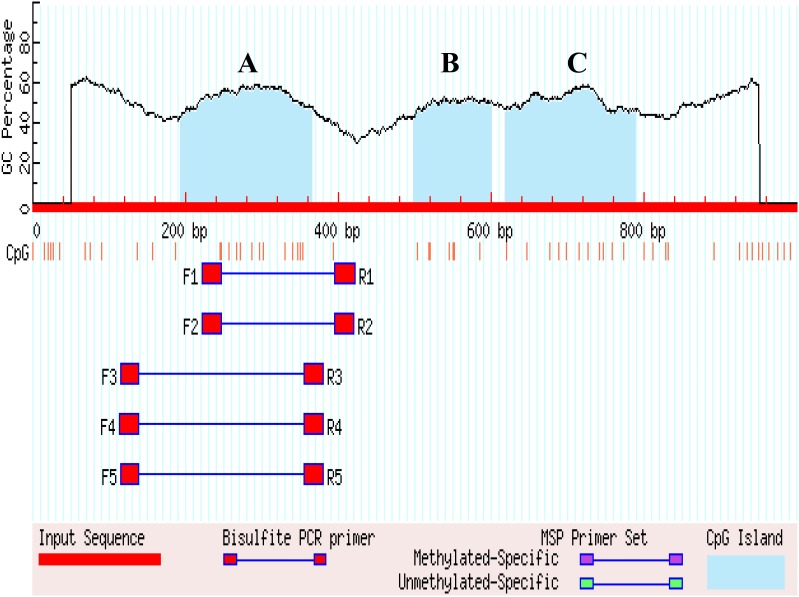
Search result graphic promoters of MSP using MethPrimer. **(A–C)** Represents the three methylated regions.

The 1 kb promoter region (the 3′ distal end 1 kb sequence of EAV-HP) was used to predict CGIs in the 4.2 kb EAV-HP insertion fragment. There are four methylated regions (A, B, and C) in the 1 kb promoter region, and five pairs primers were designed for A methylated regions (Figure 4). Using bisulfite-treated DNA samples from eggshell gland tissue as the template, PCR amplification and gel electrophoresis were used to detect primer specificity. As a result, only F1 and R1 (named Methprimer-F and Methprimer-R) had strong specificity and could serve as methylation primers (Supplementary Figure S3). Moreover, a previous report demonstrated that methylated region A is the *SLCO1B3* gene core promoter region (Wang et al., 2013).

The cytosine C base was replaced with the thymine T base in the original sequence of the *SLCO1B3* gene, and the C base of the CpG site was not included. Compared to the replaced sequence and the results of BSP, the result showed 14 CpG sites (Supplementary Figure S4). Fourteen CpG sites distributed in the core promoter region of *SLCO1B3* gene on chromosome 1 (Supplementary Figure S5). The DNA sequences from the chromatogram file were detected by Chromas software (version 2.4.1), and DNA sequencing maps revealed single peaks and no overlap (Supplementary Figure S6).

### Methylation of the *SLCO1B3* Promoter Region

**FIGURE 5:**
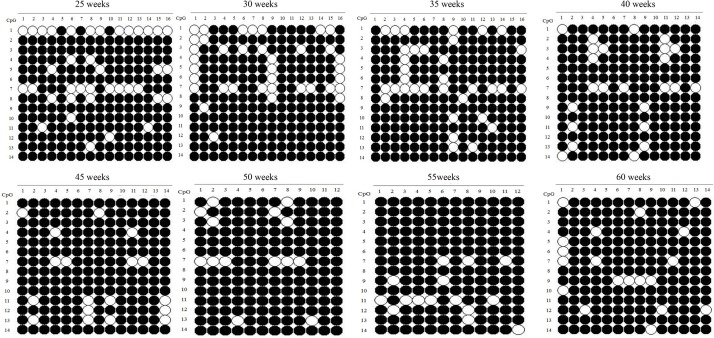
Methylation patterns of *SLCO1B3* gene promoter in the shell gland. The black dots indicate methylated, and the white dots indicate unmethylated.

**FIGURE 6:**
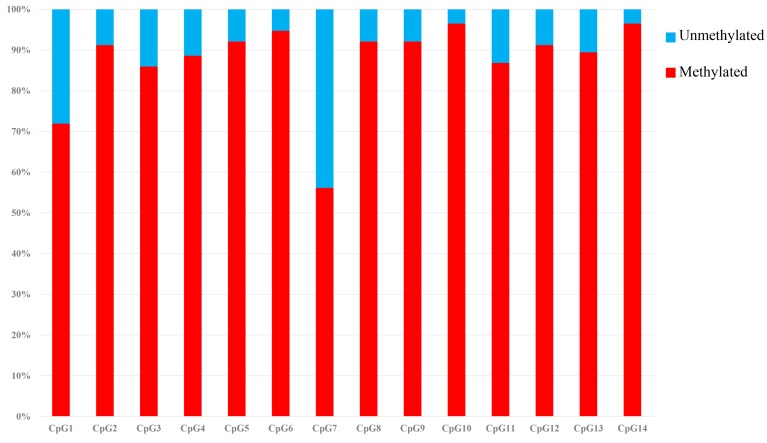
Methylation percentage of the *SLCO1B3* gene promoter region in the shell gland. The black areas indicate methylated, and the white areas indicate unmethylated.

The *SLCO1B3* gene promoter region showed a high methylation level in the chicken shell gland tissue based on the analysis of the overall methylation level, and the proportion of methylation was more than 80% in all weeks (Figure 5). The overall methylation levels of the *SLCO1B3* gene promoter during weeks 25, 30, 35, 40, 45, 50, 55, and 60 of Lushi blue eggshell chicken were 83.93, 84.37, 85.27, 87.76, 90.82, 91.67, 91.07, and 91.33%, respectively (Supplementary Table S3). In addition, the methylation rates of the 14 promoter CpG sites in the chicken shell gland tissue were 71.93, 91.23, 85.96, 88.60, 92.11, 94.74, 56.14, 92.11, 92.11, 96.49, 86.84, 91.23, 89.47, and 96.49%, respectively (Figure 6 and Supplementary Table S4).

### Correlation Analysis of *SLCO1B3* Methylation and Its Expression and Eggshell Color

The correlation analysis showed that the overall promoter methylation levels of the *SLCO1B3* gene and *SLCO1B3* expression were significantly negatively correlated in the shell gland tissue and that the correlation coefficient was -0.889 (*P* < 0.01). Furthermore, the correlation analysis of the methylation rate of the 14 promoter sites and equivalent gene expression in each week revealed that CpG1, CpG5, and CpG8 and their expression were significantly negatively correlated (*P* < 0.05); the Pearson correlation coefficient was -0.708, -0.779, -0.709, respectively (Supplementary Table S5). Moreover, there was a significant negative correlation between the overall promoter methylation level of the *SLCO1B3* gene and the eggshell color, and the correlation coefficient between the methylation level and the eggshell color value was 0.807 (*P* < 0.05). The methylation levels of the CpG1, CpG5, and CpG8 sites were significantly positively correlated with the eggshell color in each week; the correlation coefficients were 0.775 (*P* < 0.05), 0.710 (*P* < 0.05), and 0.871 (*P* < 0.01), respectively (Supplementary Table S6).

### Transcription Factors of the *SLCO1B3* Promoter Region

**FIGURE 7:**
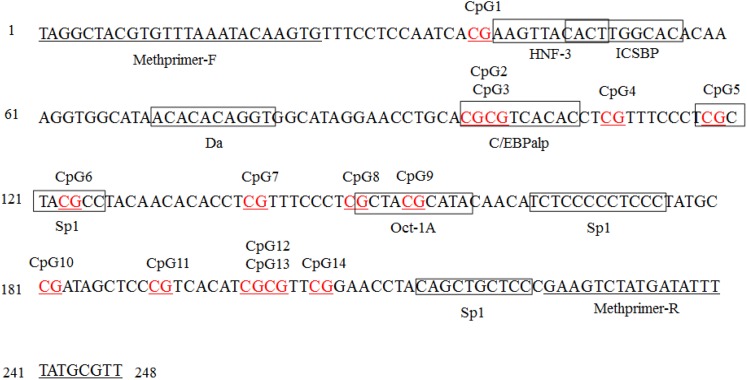
Transcription factor (TF) binding sites predicted in the *SLCO1B3* gene promoter region in the shell gland. Methprimer-F and Methprimer-R are specific methylation primers, and the sequence within each box indicates a transcription factor. The AliBaba 2.1 online tool parameters: Matwidth = 10 bp, Pairsim = 50, Cons = 75%, Promsim = 1% and Classification level *K* = 4.

The transcriptional binding sites in the *SLCO1B3* gene promoter region were analyzed by software, and the results revealed six potential transcriptional binding sites (C/EBPalp, C/EBPalp, Sp1, Sp1, Oct-1A, and Oct-1A), whose binding sites contain CpG dinucleotides CpG2, CpG3, CpG5, CpG6, CpG8, and CpG9, respectively. However, six TFs whose binding sites exclude CpG1, CpG4, CpG7, CpG10, CpG11, CpG12, CpG13, and CpG14 sites were also detected (Figure 7).

## Discussion

In birds, eggshell color is a changeable Mendelian trait, and avian eggs exhibit a major difference in coloring between species. Previous research has shown that the color change of avian eggs is associated with evolutionary biology (Kilner, 2010). The ancestral bird egg color was probably white and had no spots, and this color persists in species whose nests are not attacked by predators, while species whose nests are vulnerable to predators are more likely to lay speckled brown eggs. Interestingly, some species may have repurposed these adaptations, with, for example, blue eggshell coloration being identified by males as a sign of female quality, as well as regulating egg temperature, combating harmful solar radiation or enhancing eggshell strength (Soler et al., 2005; Reynolds et al., 2009; Kilner, 2010; Moreno and Osorno, 2010).

Unlike dinosaurs and current reptiles, avian species do not bury their eggs but usually leave them exposed to sunlight, and this process frequently accompanies avian incubation behavior, which is the major evolutionary step distinguishing birds from reptiles and is linked with the evolution of eggshell pigmentation. Shell pigments may thus be important during development of the avian embryo (Maurer et al., 2011). Studies have also shown that the pigment concentrations of birds’ eggs are phylogenetically linked to specific life-history and ecological strategies (Cassey et al., 2012). Although it is widely held that birds are the only extant amniotes producing colored eggs, protoporphyrin IX and biliverdin have both be detected in the fossilized eggshells of a non-avian dinosaur. Meanwhile, this recent study found that egg color likely evolved a single time among the non-avian theropod dinosaurs, with similar pattern diversity to that observed in modern birds (Wiemann et al., 2018). Research on poultry species has improved our understanding of eggshell pigmentation and provides new insights into the unique adaptations of egg color to environments.

Throughout the hen laying cycle, the ability of chicken eggshell glands to secrete and deposit pigments differs at different ages. Generally, young hens have just entered the sexual maturity stage, their follicles develop normally, and their eggshell gland tissue has a good ability to secrete and deposit pigments. The eggshell is often a deep color during this period. In contrast, with increases in age and the number of eggs continuously produced, the eggshell color has a tendency to gradually fade, and this phenomenon is especially obvious after 39–40 weeks of age (Sui et al., 2008). However, the molecular mechanism underlying how all types of eggshell colors gradually lighten is poorly understood to date.

In this study, there were two inflection points in the production egg period of Lushi chickens: 25 and 45 weeks. The shell color changed from dark blue to medium blue at 25 weeks of age, and at 45 weeks, the shell color changed from medium blue to light blue. The egg production rate continued to increase after 25 weeks of age, achieving peak egg production at 30 weeks of age. We suspect that the rate of production of the eggshell gland synthetic pigment is lower than the rate of egg laying during this period, causing eggshell color to gradually become lighter. The eggshell color was significantly deeper at 45 weeks of age than at 50 weeks of age (*P* < 0.05), although 50 weeks is in the later period of the laying cycle in the Chinese native chicken breed and gradual degeneration of the ability to deposit pigment might be the reason why the eggshell color was significantly lighter. A previous report showed that the chicken eggshell color became gradually lighter as egg production increased; interestingly, our results are consistent with this conclusion (Odabaşi et al., 2007). The heterozygous and homozygous forms of a gene may affect the phenotypic traits of animals to different degrees. The *CDKN2A /B* locus regulates chicken sex-linked barring, with a classical gene-dose effect observed at the B locus: B^∗^B homozygotes exhibit wider white bands than do B^∗^B/B^∗^N heterozygotes or B^∗^B/W hemizygotes (Hellström et al., 2010). Previous research has indicated that a 4.2 kb EAV-HP insertion upstream of *SLCO1B3* mediates the development of blue egg coloration among chickens. The blue eggshell color of homozygotes is a darker blue than that of heterozygotes, and expression levels of *SCLO1B3* were higher in homozygous than in heterozygous Lushi chickens (Wang et al., 2013). We suspect that the *SCLO1B3* gene has a certain dosage effect on level of gene expression and in turn affects the blue eggshell color. Based on qPCR, the *SLCO1B3* gene in blue eggshell chickens was most abundant in the eggshell gland and oviduct and lowest in the liver, while no *SLCO1B3* expression was observed in the eggshell gland and oviduct of white and brown eggshell laying hens. Fluorescence-labeled cDNA *in situ* hybridization has revealed *SLCO1B3* to be uniquely expressed in the uterine tissue of hens which lay blue eggs and absent in those that do not (David et al., 2013). These consistent results indicate that *SLCO1B3* is the relevant gene for blue eggshell color in the chicken. In this experiment, the *SLCO1B3* mRNA expression level was the highest at weeks 25 and 30, which was significantly higher than at other weeks in Lushi chicken (*P* < 0.05); expression was the lowest at weeks 50, 55, and 60. Generally, the expression level of *SLCO1B3* showed a trend of decreasing throughout the laying cycle (*P*-value = 0.36) (Figure 2). Interestingly, a strong positive correlation between the average values of eggshell color and *SLCO1B3* expression in shell glands throughout the laying cycle was shown by Pearson analysis (*P* < 0.05) (Figure 3). We hypothesize that epigenetic factors mediate altered *SLCO1B3* expression, and have conducted additional research into this hypothesis.

DNA methylation in humans is among the first detected epigenetic modifications (Jaenisch and Bird, 2003). This methylation relies upon the covalent modification of cytosine residues generally present within CpG regions, leading to alterations in gene expression including a suppression of gene expression when methylation occurs in promoter or enhancer regions (Dor and Cedar, 2018). In this study, we found that the overall methylation rate of *SLCO1B3* increased gradually with increasing weeks of egg production in Lushi chicken (Figure 5) and that the overall promoter methylation of the *SLCO1B3* gene was significantly negatively correlated with both *SLCO1B3* expression in the shell gland tissue and the color of the eggshell. Aging-related shifts in patterns of DNA methylation changes have been detected in salmon, rats, mice, and other animals (Jin and Liu, 2018). DNA methylation is a specific process in species and tissues. Overall global methylation generally increases with age, both in prokaryotic and eukaryotic organisms. Disorders in DNA methylation can lead to premature aging and control triggers of the activation of oncogenes, inactivation of neoplasm suppressor-genes and chromatin stability disorders (Bird, 2002; Vanyushin, 2005; Boks et al., 2009; Gryzińska et al., 2013). Methylation also regulates gene expression during embryogenesis, cooperating with genomic regulation to control the expression of *CDKN2B* on the 6 and 18th days of embryonic development in the chicken (Gryzińska et al., 2013).

This study is the first assessing the simultaneous genetic and epigenetic regulation of *SLCO1B3*. TFs are essential factors that tweak gene expression, and CpG methylation can disrupt TF binding via direct disruption of base recognition or via recruiting certain methylation-dependent proteins (Domcke et al., 2015). There may be many methylation sites in each CGI located in the promoter region. Specific CpG sites could possibly play a key role in the function of CGIs and in regulating gene function (Mikeska et al., 2007; Xie et al., 2012). When a methylated CpG site is found at a TF binding site, the binding efficiency of the specific binding site and the TF is inhibited, such as USF-1 and Sp1 (Aoki et al., 2008; Chuang et al., 2011). In this study, our results showed that six potential TF binding sites have three potential TFs: C/EBPalp, Sp1, and Oct-1A. The CpG1, CpG5, and CpG8 sites and *SLCO1B3* gene expression were significantly negatively correlated (*P* < 0.05); in particular, the correlation between CpG8 and gene expression was highly significant (*P* < 0.01). Further, the CpG5 and CpG8 sites had the Sp1 and Oct-1A TFs, respectively. Previous research has shown that binding of SP1 and its specific target sequence enables transcription of the starting gene and that overexpression of the TF Oct-1A caused a decrease in JAK–STAT signaling pathway gene expression, including *IFNAR2*, *STAT1*, *STAT2*, and *STAT4* (Pugh and Tjian, 1990; Richer et al., 2014; Pankratova et al., 2016). These results revealed that the CpG5 and CpG8 sites may be key sites for regulating gene transcription, while other methylation sites may be secondary sites.

## Conclusion

In summary, in the present study, the egg production period of Lushi chickens could be divided into three stages: 20 to 25 weeks of age for dark blue, 26 to 45 weeks of age for medium blue, 46 to 60 weeks of age for light blue. There are two inflection points in the production egg period of chicken, at 25 and 45 weeks, and the eggshell color gradually decreased with increasing egg production weeks. In addition, the expression level of *SLCO1B3* showed a trend of decreasing over the whole laying cycle, and a strong positive correlation was found between the average values of eggshell color and *SLCO1B3* expression in the shell glands throughout the laying cycle. Moreover, our results indicated that the overall methylation rate of the *SLCO1B3* gene in Lushi blue eggshell chickens increased gradually with increasing weeks of egg production. The promoter methylation of the *SLCO1B3* gene and *SLCO1B3* expression in the shell gland tissue were significantly negatively correlated, and there was a significant negative correlation between the promoter methylation level of the *SLCO1B3* gene and the color of the eggshell. Furthermore, we predicted that the CpG5 and CpG8 sites may be key sites for regulating gene transcription, while other methylation sites may be secondary sites. Thus, we hypothesized that as the level of methylation increases, the methylation of the CpG5 and CpG8 sites hinders the binding of the TFs to the promoter, and then the expression of the *SLCO1B3* gene is reduced in the late period, resulting in a lighter eggshell color. However, further functional studies on *SLCO1B3* are needed for confirmation. These findings will contribute to further understanding the regulatory mechanisms of *SLCO1B3* in eggshell color variations in Lushi chickens and provide useful information regarding DNA methylation and color-related traits in animals.

## Author Contributions

ZL and TR performed the measurement of eggshell color, qPCR, the prediction of CpG islands and template DNA processing, analyzed the data, and wrote the manuscript. WL and YZ collected the samples and analyzed the data. RH, HL, RJ, and FY performed the additional experiments. GS and XL revised the manuscript. YT and XK designed the study and reviewed the manuscript. All authors have read and approved the final manuscript.

## Conflict of Interest Statement

The authors declare that the research was conducted in the absence of any commercial or financial relationships that could be construed as a potential conflict of interest.

## Footnotes

1 http://www.urogene.org/methprimer

2 http://www.msrcall.com/MSRcalcalate.aspx

3 http://gene-regulation.com/pub/programs/alibaba2/index.html
