# Supplementary material for: Association Between the Methylation Statuses at CpG Sites in the Promoter Region of the SLCO1B3, RNA Expression and Color Change in Blue Eggshells in Lushi Chickens
Source: Front Genet. 2019 Feb 26;10:161. doi: 10.3389/fgene.2019.00161 (PMC6399514; doi:10.3389/fgene.2019.00161)
Supplement: Supplementary file 1 [file Data_Sheet_1.doc]

**Supplementary material**

**Association between the methylation statuses at CpG sites in the promoter region of SLCO1B3, RNA expression and color change in blue eggshells in Lushi chickens**

Zhuanjian Li^1,2,a^, Tuanhui Ren^1,a^, Wenya Li^1^, Yu Zhou^1^, Ruili Han^1,2^, Hong Li^1,2^, Ruirui Jiang^1,2^, Fengbin Yan^1,2^, Guirong Sun^1,2^, Xiaojun Liu^1,2^, Yadong Tian^1,2^*, Xiangtao Kang^1,2^*
^1^College of Animal Science and Veterinary Medicine, Henan Agricultural University, Zhengzhou, 450002, China

^2^Henan Innovative Engineering Research Center of Poultry Germplasm Resource, Zhengzhou, 450002, China


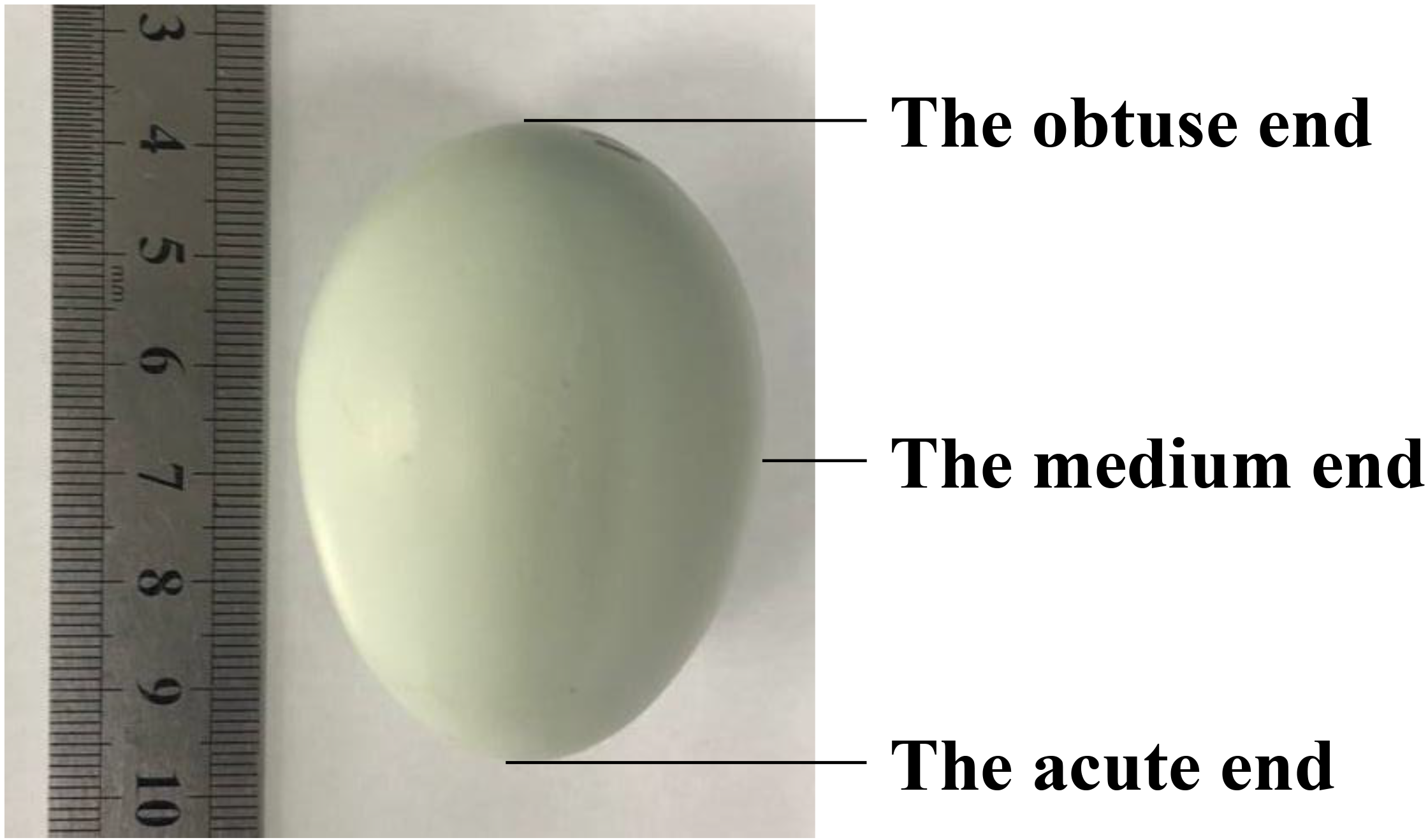


Figure S1 Schematic diagram of measurement points for the eggshell color. The picture is a photo of an egg, and we used a steel ruler to represent the scale (cm). A colorimeter (Type NR-10) for eggshell color was used for quantitative measurement of blue-shelled eggs. The colorimeter was black-and-white corrected before measuring the eggshell color every week, and then the sample was measured. Starting from the obtuse end of the egg, the obtuse, medium and acute ends of the eggshell were measured along the longitudinal axis of the egg.


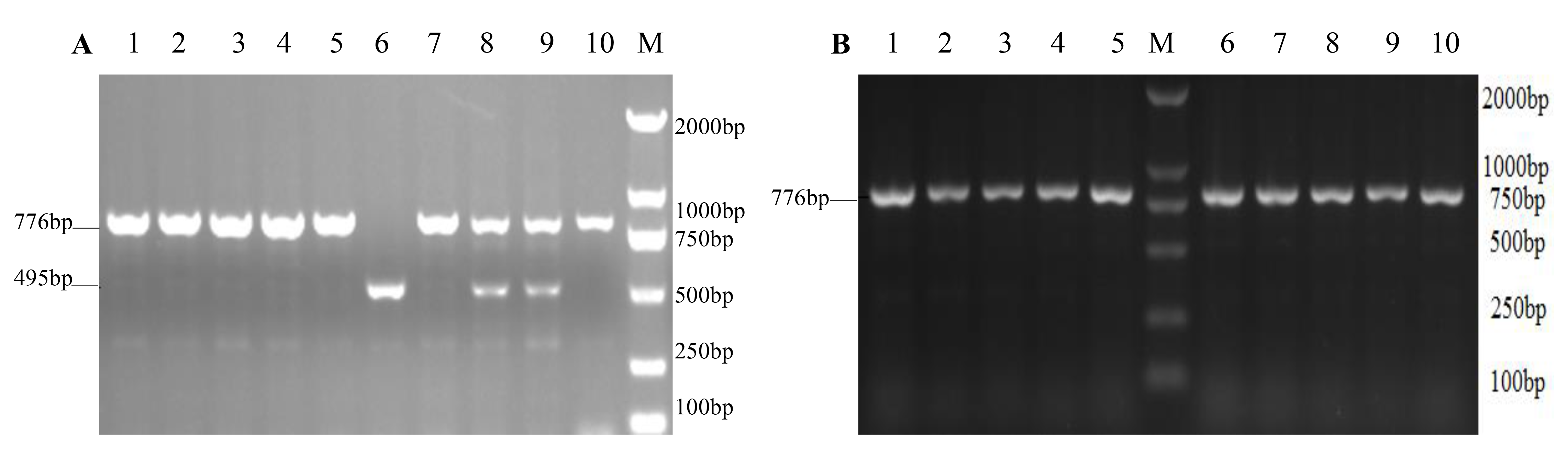


Figure S2 Agarose gel electrophoresis (1.5%) image for the *SCLO1B3* gene. (A). The genotype results of *SCLO1B3* in three chicken breeds. M represents DL 2000; 6 is the DNA sample of pink eggshell, 8 and 9 are the DNA samples of Xichuan chicken, and all the others are DNA samples of Lushi chickens. (B). The genotype results of *SCLO1B3* in shell gland tissue samples of Lushi chickens.


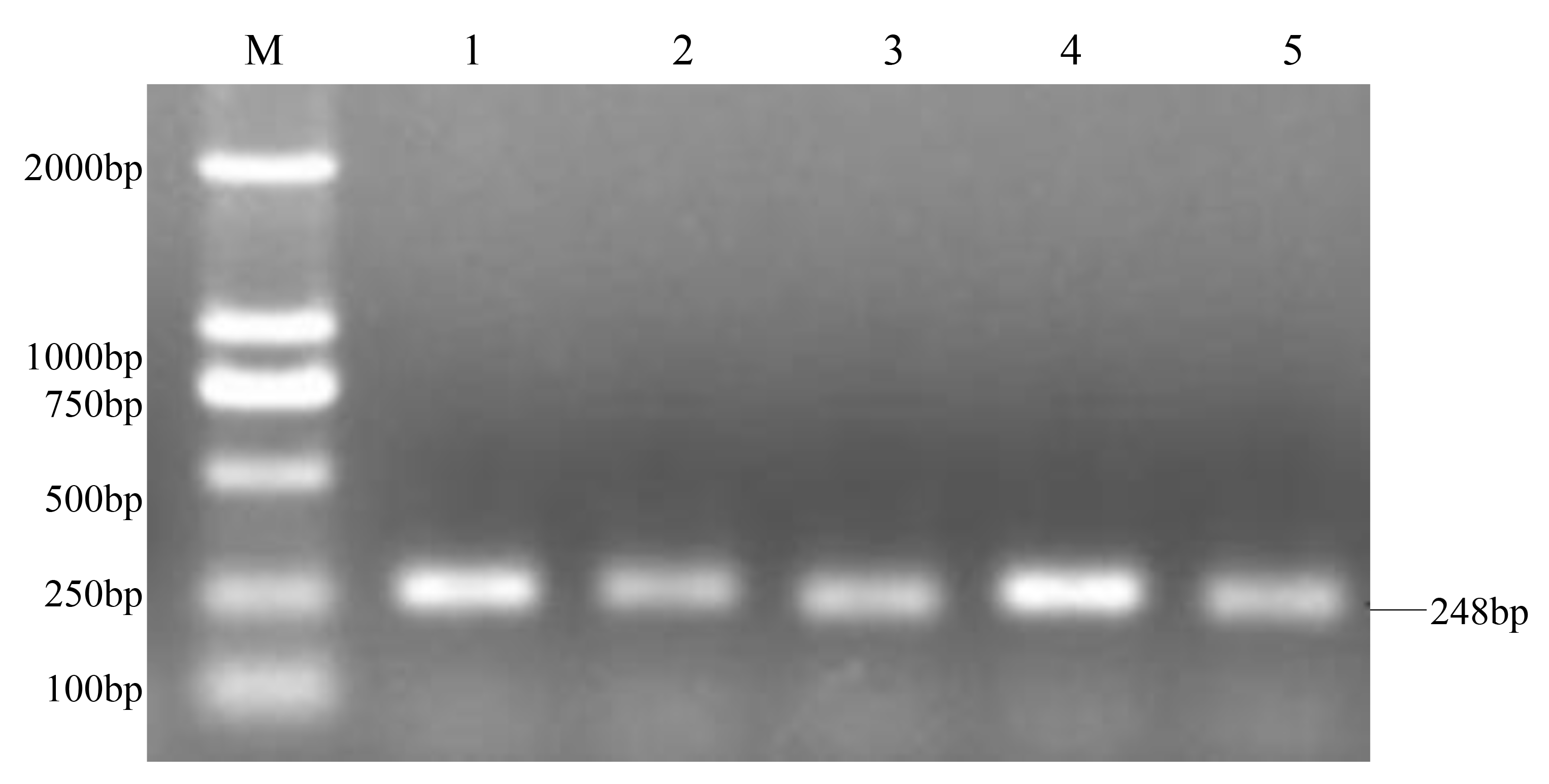


Figure S3 Agarose gel electrophoresis (1.5%) patterns showing amplified fragment length of methylation primer F1, R1. M represents DL 2000, and 1 to 5 are bisulfite-treated DNA templates from eggshell gland tissue.


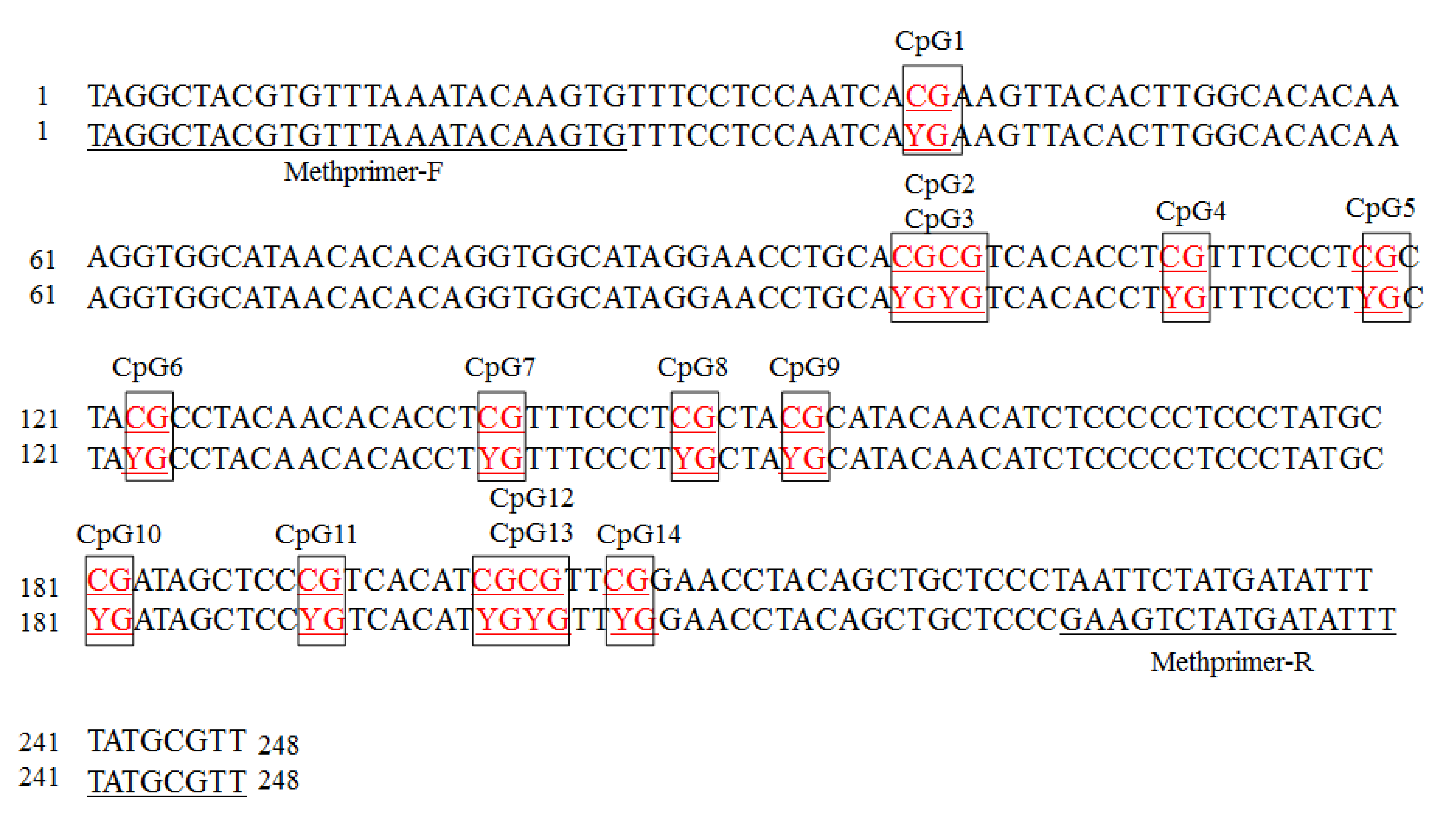


Figure S4 Methylation sequence alignment for CGIs of the *SLCO1B3* gene promoter region of Lushi blue eggshell. CG represents each CpG dinucleotide, and YG represents TG (unmethylated) or CG (methylated) in the box.


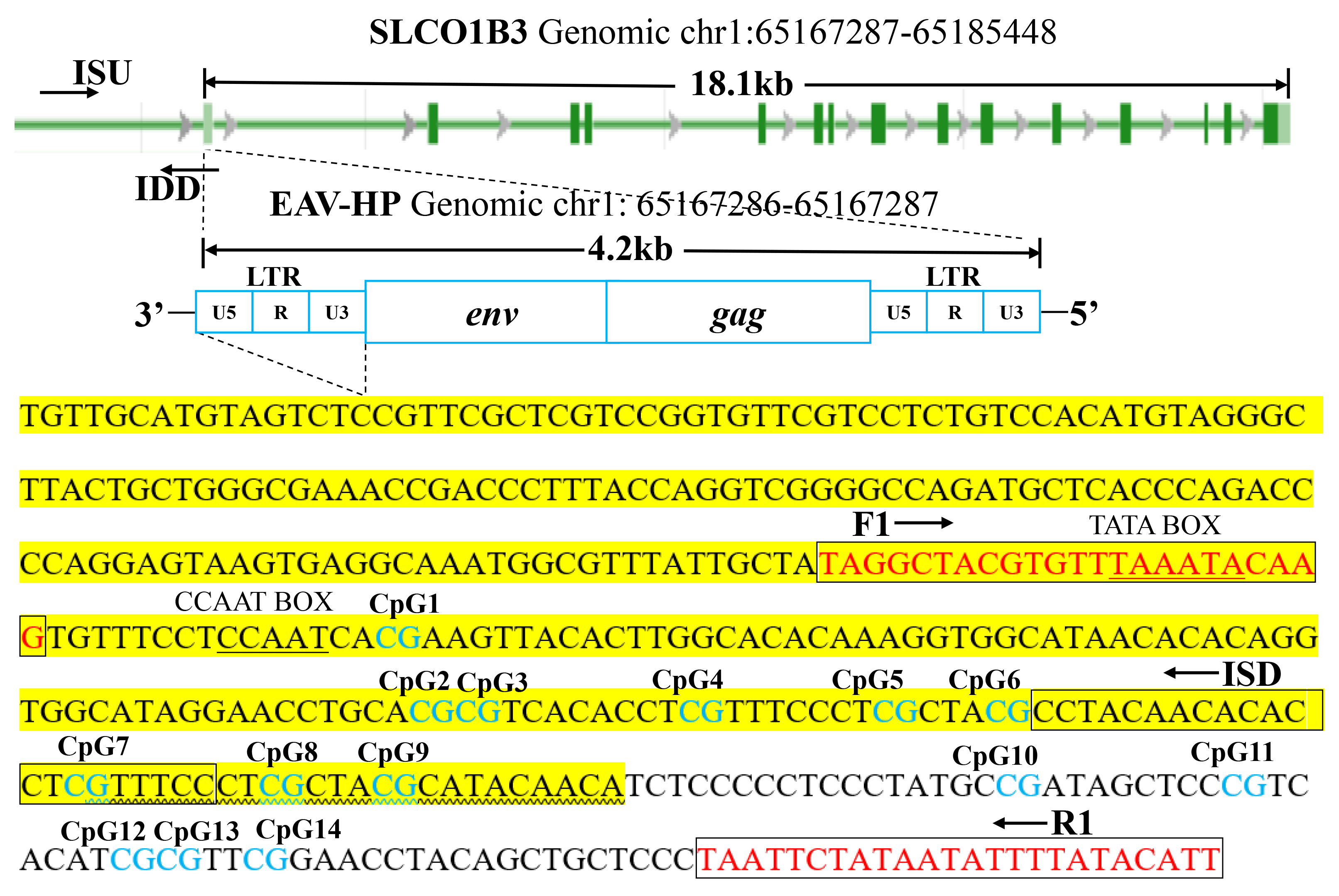


Figure S5 Relative positions of *SLCO1B3* and CpG site on chromosome 1 (Wang et al., 2013). EAV-HP insertion in the 5’ end of *SLCO1B3* in inverted orientation. A complete avian retrovirus consists of a long terminal repeat (LTR), *gag*, *pol* and *env* genes. Compared with the intact retrovirus, the EAV-HP lacks an entire pol gene and a partial sequence of the *gag* and *env*. The LTR comprises U3, R and U5, and the yellow shaded base sequence in the figure is the LTR region. The *SLCO1B3* gene core promoter region is 400 bp including the LTR at the 3’ end and a partial sequence of the *env.* The red-letter base sequence with boxes represents the methylated primers F1 and R1(Methprimer-F and Methprimer-R), the blue base represents the CpG site, and the underlined 24 bp base sequence is the specific sequence of the validated EAV-HP. When *SLCO1B3* is transcribed, an extra 24-bp sequence from the EAV-HP is also compiled into the *SLCO1B3* transcript, and the blue-shell allele containing the 24 bp EAV-HP partial sequence showed that the expression of SLCO1B3 in blue-shelled chickens is closely related to the insertion (Wang et al., 2013). ISU, IDD and ISD are the typing primers for the *SLCO1B3* gene.


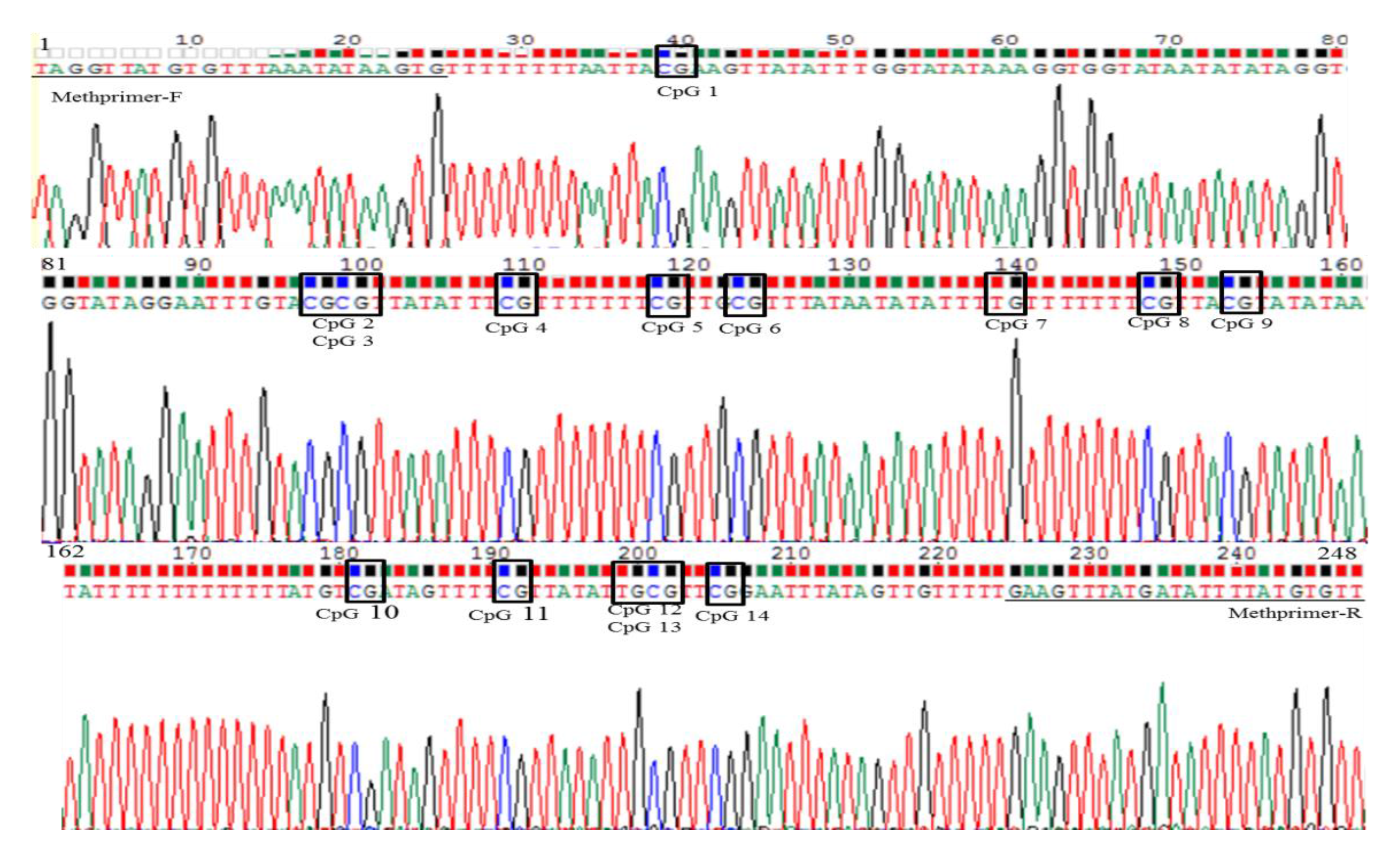


Figure S6 The Sanger sequencing chromatogram across the target sequence in the *SLCO1B3* promotor in Lushi blue eggshell chickens. The black boxes indicate the CpG or YpG dinucleotides shown in Figure S4.

Table S1 Information of the primers.

| Primer Name | Primer Sequences (5'-3') | Product Length |
| --- | --- | --- |
| F1 (Methprimer-F) | TAGGTTATGTGTTTAAATATAAGTG | 248 bp |
| R1 (Methprimer-R) | AACACATAAAATATCATAAACTTC |
| F2 | TATATAGGTGGTATAGGAATTTGTA | 200 bp |
| R2 | AACAATAAATCAATTTATAAATAAAC |
| F3 | TTTAGGAGTAAGTGAGGTAAATGG | 267 bp |
| R3 | AAAATTAAAAAACAACTATAAATTCC |
| F4 | TTTTAGGAGTAAGTGAGGTAAATGG | 268 bp |
| R4 | AAAATTAAAAAACAACTATAAATTCC |
| F5 | TTTAGGAGTAAGTGAGGTAAATGG | 266 bp |
| R5 | AAATTAAAAAACAACTATAAATTCC |
| ISU | TGTAAGAAGGCAGAGGGTTGA | 495 bp, 776 bp |
| ISD | GGAAACGAGGTGTGTTGTAGG |
| IDD | AATCTTGTCTCCTCCCAGTTC |

Note：F1, R1, F2, R2, F3, R3, F4, R4, F5, and R5 are BSP methylation primers, respectively. F1 and R1 (Methprimer-F and Methprimer-R) are a pair of specific methylation primers, and ISU, ISD and IDD are the specific detection primers for blue shell genotypes.

Table S2 The distribution of the values of eggshell color.

|  | Weeks | | | | | | | | | |
| --- | --- | --- | --- | --- | --- | --- | --- | --- | --- | --- |
| Eggshell color | 20 | 25 | 30 | 35 | 40 | 45 | 50 | 55 | 60 | Total |
| ＜50 | 38 | 12 | 22 | 3 | 9 | 12 | 0 | 0 | 0 | 96 |
| 50- | 33 | 80 | 11 | 15 | 16 | 20 | 5 | 13 | 3 | 196 |
| 52- | 61 | 99 | 33 | 33 | 29 | 60 | 19 | 37 | 18 | 389 |
| 54- | 72 | 72 | 64 | 72 | 47 | 42 | 42 | 51 | 25 | 487 |
| 56- | 60 | 50 | 92 | 75 | 74 | 66 | 80 | 62 | 32 | 591 |
| 58- | 0 | 22 | 71 | 54 | 60 | 44 | 95 | 67 | 46 | 459 |
| 60- | 0 | 2 | 17 | 28 | 33 | 16 | 59 | 38 | 30 | 223 |
| 62- | 0 | 0 | 3 | 10 | 17 | 59 | 48 | 45 | 30 | 212 |
| Total | 264 | 337 | 313 | 290 | 285 | 319 | 348 | 313 | 184 | 2653 |

Note: The underlined value represents the maximum value of the eggshell color that occurred at each week of age.

Table S3 Methylation analysis of the 5' flanking sequence of the *SLCO1B3* gene in the shell gland of Lushi blue eggshell chickens in different weeks.

| Week | Number of Methylation Site | Number of Successful Sequencing | Methylation Rate |
| --- | --- | --- | --- |
|
| 25 | 188 | 16 | 83.93% |
| 30 | 182 | 16 | 84.37% |
| 35 | 191 | 16 | 85.27% |
| 40 | 172 | 14 | 87.76% |
| 45 | 178 | 14 | 90.82% |
| 50 | 154 | 12 | 91.67% |
| 55 | 139 | 12 | 91.07% |
| 60 | 174 | 14 | 91.33% |

Note：Methylation Rate refers to the overall methylation across all CpG sites successfully sequenced. Number of Methylation Site refers to the total amount of Methylation site in 12-16 positive clones each week.

Table S4 Methylation percentage of the *SLCO1B3* gene promoter region in the shell gland.

| CpG Site | Methylation Percentage (%) |
| --- | --- |
|
| CpG1 | 71.93 |
| CpG2 | 91.23 |
| CpG3 | 85.96 |
| CpG4 | 88.60 |
| CpG5 | 92.11 |
| CpG6 | 94.74 |
| CpG7 | 56.14 |
| CpG8 | 92.11 |
| CpG9 | 92.11 |
| CpG10 | 96.49 |
| CpG11 | 86.84 |
| CpG12 | 91.23 |
| CpG13 | 89.47 |
| CpG14 | 96.49 |

Table S5 Association analysis between the expression of the *SLCO1B3* gene and the methylation level of the gene in different egg-producing periods.

|  |  | Methylation rate at the CpG target sites | | | | | | | | | | | | | | |
| --- | --- | --- | --- | --- | --- | --- | --- | --- | --- | --- | --- | --- | --- | --- | --- | --- |
| Weeks | Expression | CpG1 | CpG2 | CpG3 | CpG4 | CpG5 | CpG6 | CpG7 | CpG8 | CpG9 | CpG10 | CpG11 | CpG12 | CpG13 | CpG14 | Whole |
| 25 | 1.4085 | 0.188 | 1 | 1 | 0.875 | 0.75 | 1 | 0.438 | 0.688 | 1 | 0.938 | 0.875 | 0.938 | 0.938 | 1 | 0.839 |
| 30 | 1.8561 | 0.563 | 0.875 | 0.625 | 0.813 | 0.813 | 0.813 | 0.438 | 0.875 | 0.938 | 1 | 1 | 0.938 | 1 | 1 | 0.844 |
| 35 | 1.2813 | 0.625 | 0.938 | 0.75 | 0.875 | 0.938 | 0.875 | 0.375 | 0.875 | 1 | 0.875 | 0.938 | 0.938 | 0.875 | 1 | 0.853 |
| 40 | 0.8749 | 0.857 | 0.857 | 0.714 | 0.857 | 1 | 1 | 0.571 | 1 | 0.857 | 1 | 0.857 | 1 | 0.857 | 0.857 | 0.878 |
| 45 | 0.9577 | 1 | 0.857 | 1 | 0.857 | 1 | 1 | 0.714 | 1 | 1 | 1 | 0.714 | 0.857 | 0.714 | 1 | 0.908 |
| 50 | 0.6945 | 0.833 | 0.833 | 0.833 | 1 | 1 | 1 | 0.5 | 1 | 1 | 1 | 1 | 1 | 0.833 | 1 | 0.917 |
| 55 | 0.7589 | 1 | 1 | 1 | 1 | 1 | 1 | 0.75 | 1 | 0.833 | 1 | 0.5 | 0.833 | 0.917 | 0.917 | 0.911 |
| 60 | 0.6752 | 0.857 | 0.929 | 1 | 0.857 | 0.929 | 0.929 | 0.786 | 1 | 0.643 | 0.929 | 1 | 0.786 | 1 | 0.929 | 0.913 |
| R-value | - | -0.708* | 0.097 | -0.523 | -0.597 | -0.779* | -0.693 | -0.69 | -0.709* | 0.449 | -0.15 | 0.297 | 0.282 | 0.344 | 0.495 | -0.889** |
| P-value | - | 0.049 | 0.82 | 0.184 | 0.118 | 0.023 | 0.057 | 0.058 | 0.049 | 0.265 | 0.723 | 0.475 | 0.499 | 0.403 | 0.212 | 0.003 |

Note: “–” corresponds to no effective value，**P* ≤ 0.05; ***P* ≤ 0.01.

Table S6 Association analysis between the measurement value for the eggshell color and the methylation level of the CpG target sites in the SLCO1B3 gene promotor in different egg-producing periods.

|  |  | Methylation rate at the CpG target sites | | | | | | | | | | | | | | |
| --- | --- | --- | --- | --- | --- | --- | --- | --- | --- | --- | --- | --- | --- | --- | --- | --- |
| Weeks | color | CpG1 | CpG2 | CpG3 | CpG4 | CpG5 | CpG6 | CpG7 | CpG8 | CpG9 | CpG10 | CpG11 | CpG12 | CpG13 | CpG14 | Whole |
| 25 | 53.5 | 0.188 | 1 | 1 | 0.875 | 0.75 | 1 | 0.438 | 0.688 | 1 | 0.938 | 0.875 | 0.938 | 0.938 | 1 | 0.839 |
| 30 | 56.31 | 0.563 | 0.875 | 0.625 | 0.813 | 0.813 | 0.813 | 0.438 | 0.875 | 0.938 | 1 | 1 | 0.938 | 1 | 1 | 0.844 |
| 35 | 56.25 | 0.625 | 0.938 | 0.75 | 0.875 | 0.938 | 0.875 | 0.375 | 0.875 | 1 | 0.875 | 0.938 | 0.938 | 0.875 | 1 | 0.853 |
| 40 | 56.78 | 0.857 | 0.857 | 0.714 | 0.857 | 1 | 1 | 0.571 | 1 | 0.857 | 1 | 0.857 | 1 | 0.857 | 0.857 | 0.878 |
| 45 | 56.46 | 1 | 0.857 | 1 | 0.857 | 1 | 1 | 0.714 | 1 | 1 | 1 | 0.714 | 0.857 | 0.714 | 1 | 0.908 |
| 50 | 58.8 | 0.833 | 0.833 | 0.833 | 1 | 1 | 1 | 0.5 | 1 | 1 | 1 | 1 | 1 | 0.833 | 1 | 0.917 |
| 55 | 57.56 | 1 | 1 | 1 | 1 | 1 | 1 | 0.75 | 1 | 0.833 | 1 | 0.5 | 0.833 | 0.917 | 0.917 | 0.911 |
| 60 | 58.74 | 0.857 | 0.929 | 1 | 0.857 | 0.929 | 0.929 | 0.786 | 1 | 0.643 | 0.929 | 1 | 0.786 | 1 | 0.929 | 0.913 |
| *R*-value | - | 0.775^*^ | -0.421 | -0.004 | 0.423 | 0.710^*^ | 0.026 | 0.519 | 0.871^**^ | -0.524 | 0.24 | 0.081 | -0.242 | -0.019 | -0.305 | 0.807^*^ |
| *P*-value | - | 0.024 | 0.3 | 0.993 | 0.296 | 0.048 | 0.952 | 0.188 | 0.005 | 0.183 | 0.567 | 0.85 | 0.564 | 0.964 | 0.462 | 0.016 |

Note: “–” corresponds to no effective value，**P* ≤ 0.05; ***P* ≤ 0.01.
